# Efficient crop straws biotreatment using the fungus *Cerrena Unicolor* GC.u01

**DOI:** 10.1186/s13568-024-01668-6

**Published:** 2024-02-24

**Authors:** Wang Ying, Cai Chunjing, Lu Junhua, Li Xuan, Wang Zhaojiang, Chu Jie

**Affiliations:** 1 Biology Institute, Qilu University of Technology (Shandong Academy of Sciences), Ji’nan, Shandong 250103 China; 2 State Key Laboratory of Biobased Material and Green Papermaking, Qilu University of Technology, Shandong Academy of Sciences, Jinan, Shandong 250353 China

**Keywords:** *Cerrena Unicolor*, Laccase, Biodegradation, Lignin, Agricultural straws

## Abstract

Lignin is main composition of agricultural biomass which can be decomposed through enzymatic hydrolysis by fungi. However, there are still needs to identify more efficient and effective fungal stain for biomass valorization. In this study, lignin degrading fungi from birch forest were screened for sustainable degradation of waste agricultural straws. The most effective strain was identified as *Cerrena unicolor* GC.u01 using 18 S rDNA gene-sequencing technology. Three different crop straws (corn stalk, rice and wheat straws) were used for the biotreatment studies. The activities of lignin degrading enzymes, laccase (Lac), cellulase and xylanase, secreted by *C. unicolor* were also determined. Scanning electron microscopy (SEM), fourier transform infrared spectroscopy (FTIR) and thermal gravimetric analyzer (TGA) were further used to monitor the effects of the biotreatment process. The results showed that *C. unicolor* degraded 34.3% rice straw lignin, a percentage which was higher than other isolated strains after 15 d straw liquid fermentation. The highest Lac activity (8.396 U•mL^− 1^) was observed with corn stalk on the 7 d. Cellulase and xylanase activities, in the same biomass, were higher than those of wheat and rice straws after 15 d. Furthermore, SEM, FTIR and TGA analyses showed that *C. unicolor* pretreatment process had significant effects on corn stalk, rice and wheat straws’ structures. The newly isolated stain of *C. unicolor* demonstrated high lignin degradation potential that can provide effective, ecofriendly means of valorizing biomass to industrial useable raw-material.

## Introduction

Agricultural straws, as a kind of carbohydrate-rich raw material, is mainly composed of cellulose, hemicellulose and lignin, which can be used as raw material of high value-added products (Mei et al. 2020). Lignin is a phenylpropanoid biopolymer, gives mechanical strength to the plant structure. Cellulose and hemicellulose make up the entire biomass and are firmly connected to the lignin molecules via covalent and hydrogenic linkages which make the structure extremely strong. Therefore, the degradation of lignin is usually the key obstacle for straw utilization. Several studies have proved that pretreatments work by deconstructing the lignin structure, thereby increasing enzymes accessibility to cellulose in the biomass (Mustafa et al. 2016; Corrêa et al. 2020).

Currently, the biological pretreatment of lignocellulosic biomass materials has received several research attentions because it offers many advantages, such as cost-effectiveness, eco-friendliness and high propensity in degrading lignocellulosic materials. Many microbial species are capable of degrading lignin and make the plant biomass more amenable to saccharification (Qian et al. 2020). The fungi and bacteria significantly occupy vital role in the metabolic systems for the degradation of lignin during biomass processing. In the process of lignin degradation, fungi have better degradation ability than bacteria. The degradation rate of some high quality strains could reach 23.6 ~ 33.4% but need more time (20 d) (Mustafa et al. 2016; Mishra et al. 2017). The degradation and mineralisation of lignin are almost exclusive to white rot fungi. The property of selective white rot fungi to degrade the lignin compound of wood in preference to cellulose and hemicelluloses may be useful. It has been shown that the pretreatment of wood chips with selective white rot fungi leads to energy savings of about 30% (Scott et al. 2002). Nowadays, large screening programs have been carried out to find the most adequate strains especially white rot fungi for industrial applications where high efficiency are sought (Zhao et al. 2020; Méndez-Hernández et al. 2019). However, their degradation efficiency is not high enough presently, and we need to find a strain that can degrade lignin more efficiently and faster.

On the other hand, many researchers have studied on the catalytic mechanism of lignin degradation by the enzyme system secreted by white rot fungi. While growing on wood, white rot fungi produce extracellular laccase (Lac, EC1.10.3.2), lignin peroxidase (LiP, EC1.11.1.14) and manganese peroxidase (MnP, EC1.11.1.13) due to the large structure of lignin polymers (Chen et al. 2012; Cui et al. 2021). Also the degrading enzymes take oxidation reaction rather than hydrolysis to achieve the aim of loosening lignin. To a large extent, the lignin degrading enzyme system is non-specific. Some white rot bacteria produce all these three enzymes, while most white rot fungi produce only two or even one (Hatakka, 1994; Eggert et al. 1996a, b), thus indicating the presence of these three enzymes is not all required. However, there is always at least MnP or Lac for lignin degradation and LiP is less common. Of the three mentioned enzymes, Lac belongs to the copper-containing oxidase family, which can be produced by several plants, insects, fungi and bacteria. Fungal Lac has higher redox potential compared to those derived from bacteria and plants, thus the fungal enzyme found more applications in lignin degradation. Nowadays, Lac has been employed for various environmental applications including decolorization, lignin degradation, bioenergy production, bioremediation and so on. Several researches have been conducted in Lac production by fungi fermentation (Yang et al. 2017). Zimbardi et al. optimized the fermentation conditions and improved the Lac yield of *Pycnoporus sanguineus* RP15 to 138.6 U·g^· 1^ using wheat bran and corncob (Zimbardi et al. 2016). Various substrates also showed effect on Lac activity, *Trichoderma harzianum* HZN10 secreted maximum Lac activity of 65 U·g^· 1^ using wheat bran (Bagewadi et al. 2017). Simultaneously, there were significant differences in Lac activity among strains, finding the potential Lac producer microorganism is still relevant for customization of Lac production.

In this research, three different crop straws (corn stalk, rice and wheat straws) were selected as the research material. We aimed to screen a kind of strain with degradation properties for lignin from nature to make full use of the agricultural straws being discarded as waste. The screened strain was identified as *Cerrena unicolor* GC.u01 by 18 S rDNA gene-sequencing technology. And the production performances of related enzyme secreted by GC.u01 were determined. The effect of GC.u01 on the structure and composition of crop straws during the process of pretreatment were studied. The strain can degrade lignin more effectively and faster, and has potential application in biomass utilization and lignin control. It can enrich the species degrading lignin, providing alternative strains for lignin degradation and utilization.

## Materials and methods

### Enrichment and identification of fungus strain

The enrichment medium for fungi composed of potato 200 g•L^− 1^, glucose 20 g•L^− 1^, KH_2_PO_4_ 3 g•L^− 1^, MgSO_4_∙7H_2_O 1 g•L^− 1^, Vitamin B1 0.02 g•L^− 1^, agar 16 g•L^− 1^, and the initial pH was neutral. The medium components for fermentative enzyme production were yeast extract 2 g•L^− 1^, NaNO_3_ 3 g•L^− 1^, KH_2_PO_4_ 0.8 g•L^− 1^, K_2_HPO_4_ 0.2 g•L^− 1^, MgSO_4_·7H_2_O 0.5 g•L^− 1^, MnSO_4_ 0.034 g•L^− 1^, CuSO_4_·5H_2_O 0.0125 g•L^− 1^ and the initial pH were 5.2 for corn stalk, 6.0 for wheat straw and 4.5 for rice straw. The guaiacol medium components were guaiacol 0.4mL•L^− 1^, yeast extract 10 g•L^− 1^, glucose 20 g•L^− 1^, agar 18 g•L^− 1^. The aniline blue medium components were aniline blue 0.1 g•L^− 1^, yeast extract 10 g•L^− 1^, glucose 20 g•L^− 1^, agar 18 g•L^− 1^. The manganese medium components were aniline blue 0.1 g•L^− 1^, yeast extract 10 g•L^− 1^, glucose 20 g•L^− 1^, agar 18 g•L^− 1^. The crop straws were collected in the farmland of Ji’nan. Solid soil sample (1 g) collected from birch forest of Ji’nan was dissolved with 10 mL sterilized redistilled water for 24 h. Leaching solution was diluted 10, 100 and 1000 times before cultured on potato dextrose agar (PDA) plate. When the fungi appeared, different strains were isolated, and repeated plate streaking was carried out at 28 °C for about 3 days until purified isolates were obtained (Wang et al. 2018). The purified colony was cultured on guaiacol, aniline blue and manganese medium, respectively.

The genomic DNA of strain GC.u01 was extracted using E.Z.N.A. Fungal DNA Kit (D3390-01, OMEGA bio-tek). The 18 S rRNA gene was amplified from the genome DNA via PCR using the primers F and R (F-GTAGTCATATGCTTGTCTC, Re-TCCGCAGGTTCACCTACGGA). Primer synthesis was completed by BGI TECH SOLUTIONS (BEIJING LIUHE) CO., LIMITED. The polymerase chain reaction (PCR) was performed with PCR mix kit (2×pfu Master mix, GK8008, Shanghai Generay Biotech Co., Ltd). The PCR product was ligated to the pMD19-T vector (TaKaRa, kusatsu, Japan) and sequenced by Sangon Biotech Co., Ltd. The obtained partial 18 S rDNA sequence data was submitted to GenBank database to make an alignment (Access number: MW150799.1). The sequence of the 18 S rRNA gene was compared with those in the GenBank https://blast.ncbi.nlm.nih.gov/Blast.cgi; accessed on 10 September 2023) databases using BLASTN. The phylogenetic analysis was performed by MEGA 7.0 using the neighbor-joining method (N-J) (Auckland, New Zealand) (Kumar et al. 2016). The bootstrap values of each branch were tested by 1000 repetitions.

The mycelium (1 cm*1 cm) on the solid media was transferred into 50 mL liquid fermentation medium and cultured at 28 °C. Eight bottles of fermentation mediums were prepared. After fermented for different days (0, 1, 2, 3, 4, 5, 6, 7 d), the mass of dried mycelium in these culture mediums were weighted. One bottle of dried mycelium was measured per day (0, 1, 2, 3, 4, 5, 6, 7 d). The growth curve of GC.u01 was plotted and analyzed.

### Measurement of ligninolytic enzymes and data analysis

For the fermentation experiment, the purified isolation GC.u01 was inoculated onto 250 mL conical flask with 50 mL liquid fermentation medium and 2.15 g stalks (corn stalk, rice and wheat straws). All the stalks were from farmland around Ji’nan. The fermentation was carried out at 28 °C in a rotary shaker (150 rpm). The experiments were done in triplicates.

For enzyme activity assay, the fermented samples were centrifuged at 6000 rpm for 10 min to remove suspended solids. Then the supernatant (crude sample of the enzyme) was used to detect Lac activity by monitoring the oxidation of 2,2’-azino-bis (3-ethylbenzthiazoline-6-sulfonic acid) (ABTS) at 420 nm (One enzyme activity unit was the required amount of Lac to convert one µmol substrate per minute) (Niladevi et al. 2008). MnP activity was measured using 2,6-dimethoxyphenol as the substrate at 469 nm (One enzyme activity unit was the required amount of MnP to convert one µmol substrate per minute) (Field et al. 1992). LiP activity was measured using resveratrol as the substrate at 310 nm (One enzyme activity unit was the required amount of LiP to convert one µmol substrate per minute) (Arora et al. 2001). Cellulase activity was measured using filter paper as the substrate at 520 nm (One enzyme activity unit was the required amount of enzyme to produce one µg glucose per minute) (Corrêa et al. 2020). Xylanase activity was measured using xylan as the substrate at 520 nm (One enzyme activity unit was the required amount of enzyme to produce one µg xylose per minute) (Corrêa et al. 2020).

The activities of Lac, MnP and LiP were calculated as Eq. (1) (Mei et al. 2020).

1 $$U/T = \frac{{\Delta OD}}{{t*\varepsilon }}*\frac{{Vt}}{{Vs}}*{10^6}$$

Where the *Vt* and *Vs* represent the total volume of measurement system (3 mL) and volume of supernatant added to the reaction (0.15 mL), respectively. Reaction time *t* is 1 min.

$$Lac:\varepsilon 420 = 36000$$

$$MnP:\varepsilon 469 = 8100$$

$$LiP:\varepsilon 310 = 9300$$

## Chemical components analyses of straw before and after fermentation

The crop straws (corn stalk, rice and wheat straws) were collected from surrounding farmland of Ji’nan. The dried crop straws were crushed and the samples passed through 20 meshes were used to ferment. The culture medium was sterilized at 121 °C for 30 min after fermented, and the filtered residue was collected and dried to constant weight at 55 °C. According to previous report (Van et al. 1991), the contents of hemicellulose, cellulose and lignin of 15 days-fermented stalks were determined by differential weight method, and the unfermented stalks was used as control to calculate the degradation rate of lignin.

The lignin percentage degradation (PD) was calculated by Eq. (2).

2 $$PD\left( \% \right) = [({L_0} - {L_a})/{L_0}] \times 100$$

Where *L*_*0*_ means the lignin content in un-fermented stalks and *L*_*a*_ means the lignin content in stalks treated with GC.u01.

## SEM, FTIR and TGA analyses

The mycelium in the treated straws was washed away and the filtered residue of straws was collected. The detailed morphological analysis of treated and untreated straws was carried out using SEM (Zeiss, SUPRA 55, Germany). The functional groups of characteristics of treated and untreated straws were examined by a VERTEX70 FTIR spectroscope (Bruker, GER). Samples were prepared by KBr pelleting. The spectra were recorded using 32 scans per sample at 4 cm^− 1^ resolution in the range of 400–4000 cm^− 1^. Thermal properties of treated and untreated straws were measured using TGA/DSC3 + thermal analyzer (METTLER TOLEDO, Swit) from room temperature to 800 °C under a nitrogen environment at a heating rate of 10 °C/min.

## Results

### Screening and identification of GC.u01

**Fig. 1:**
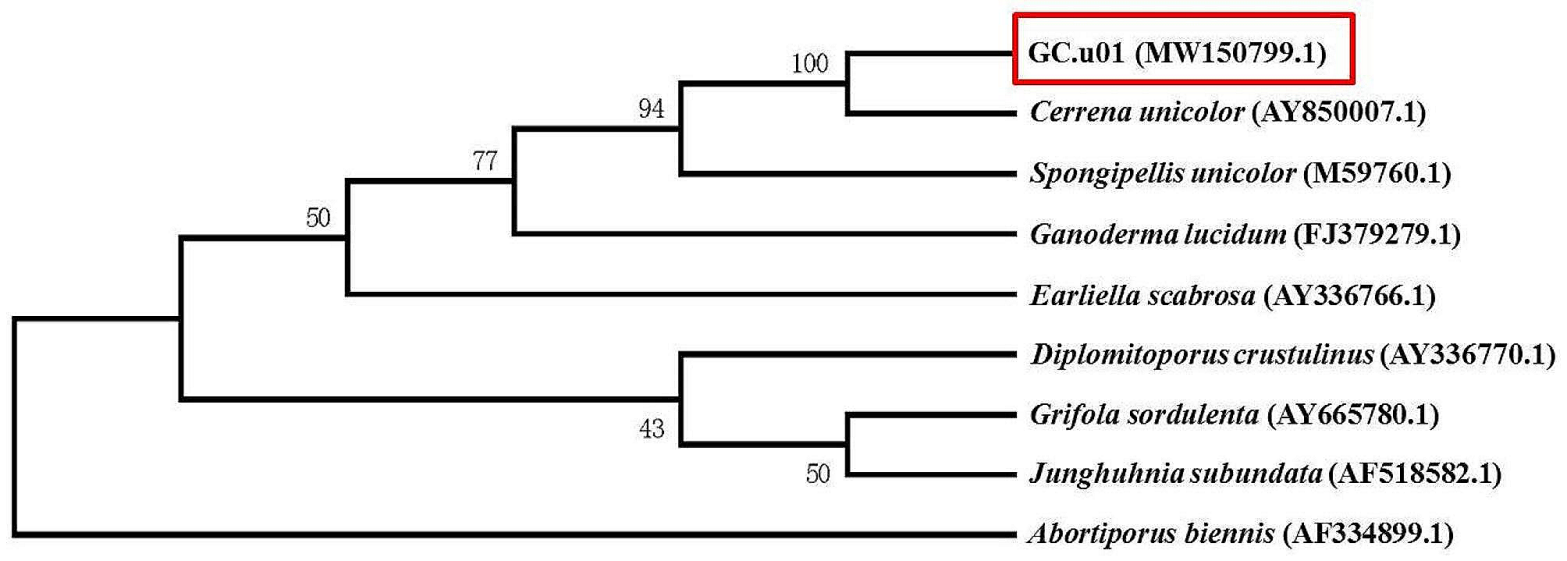
Phylogenetic tree of GC.u01 based on the 18 S rDNA sequences. The top eight most similarity strains were chosen in the GenBank databases using BLASTN

After three-day cultivation, an isolate strain was found to produce halos on guaiacol, aniline blue and manganese mediums. The purified strain was named GC.u01. To identify strain GC.u01, the 18 S rDNA gene (1699 bp) of GC.u01 was amplified and sequenced. The sequence of the 18 S rRNA gene was compared with those in the GenBank databases using BLASTN. A phylogenetic analysis was performed using the N-J method. The result showed that strain GC.u01 (MW150799.1) was most close to *Cerrena unicolor* (AY850007.1) (Fig. 1). It suggested that GC.u01 belongs to the same species of *Cerrena unicolor*. GC.u01 was stored in China General Microbiological Culture Collection Center (CGMCC), and numbered as CGMCC 40,863 (http://www.cgmcc.net).

**Fig. 2:**
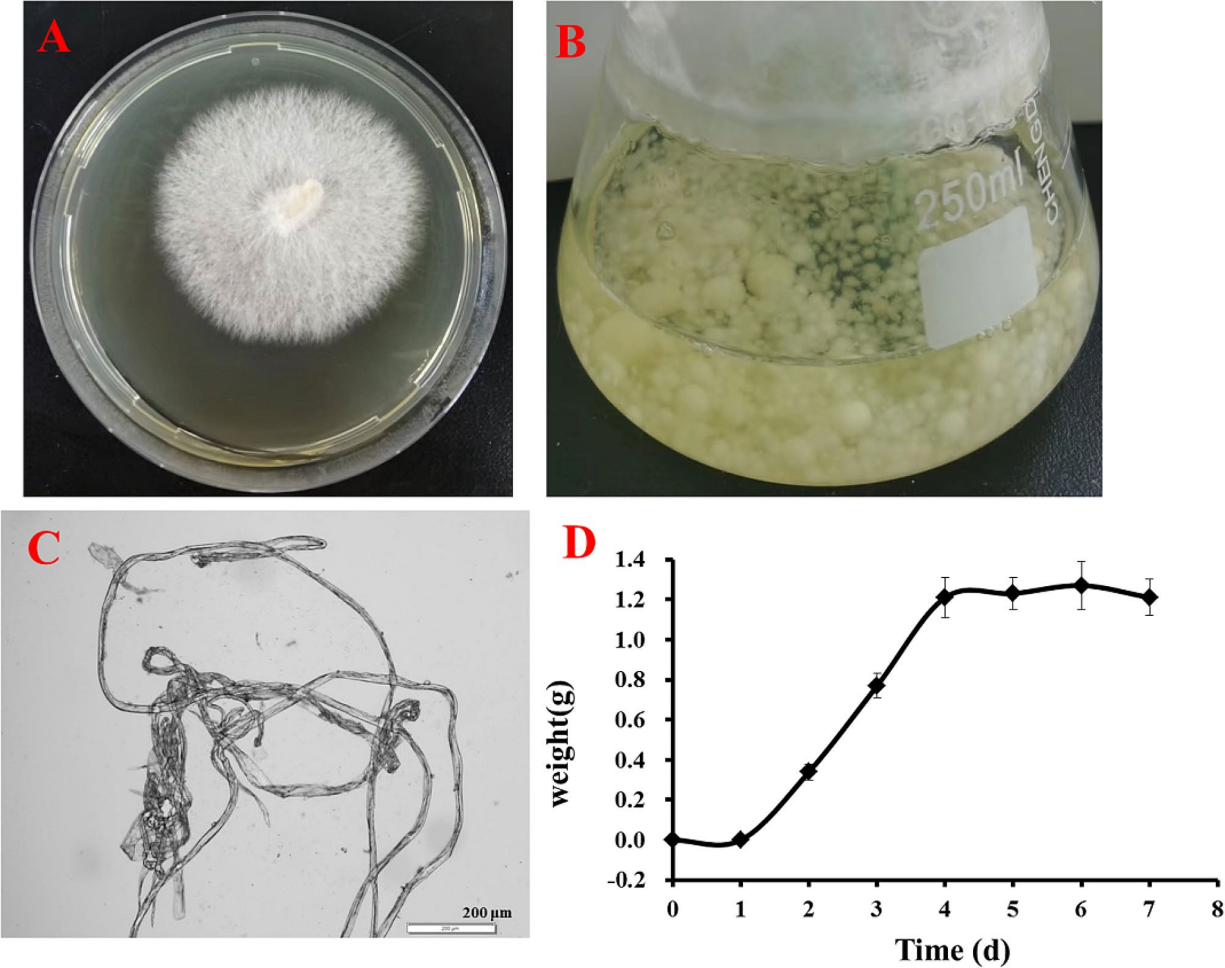
Morphological characteristics of GC.u01. **(A)** colony on PDA plate; **(B)** pellets in liquid medium; **(C)** hyphae morphology of colony magnified 100 times; **(D)** growth curve

The *Cerrena unicolor* GC.u01 was cultured on PDA plate medium and the colony morphology was observed during the growth process. The mycelia were diverged white floc, and grew outward at the initial growth stage. The colony was approximately irregular and round. The conidia appeared after five days of growth on the plate and mycelium was short and messy as catkin fluffy (Fig. 2A). After ten days of growth, the colony showed concentric wheel striations, and light beige emission folds on the back of the colony. With the increased incubation time of GC.u01 in liquid medium, many small white pellets of even size grew from the original pellets (Fig. 2B). The hyphae morphology of GC.u01 showed filaments with branches under electron microscope (Fig. 2C).

The growth curve of GC.u01 was measured. It could be seen that GC.u01 enters the logarithmic growth period after one day and it entered the stable phase on the 4th day (Fig. 2D).

### Process of enzyme production via submerged fermentation of crop straws

**Fig. 3:**
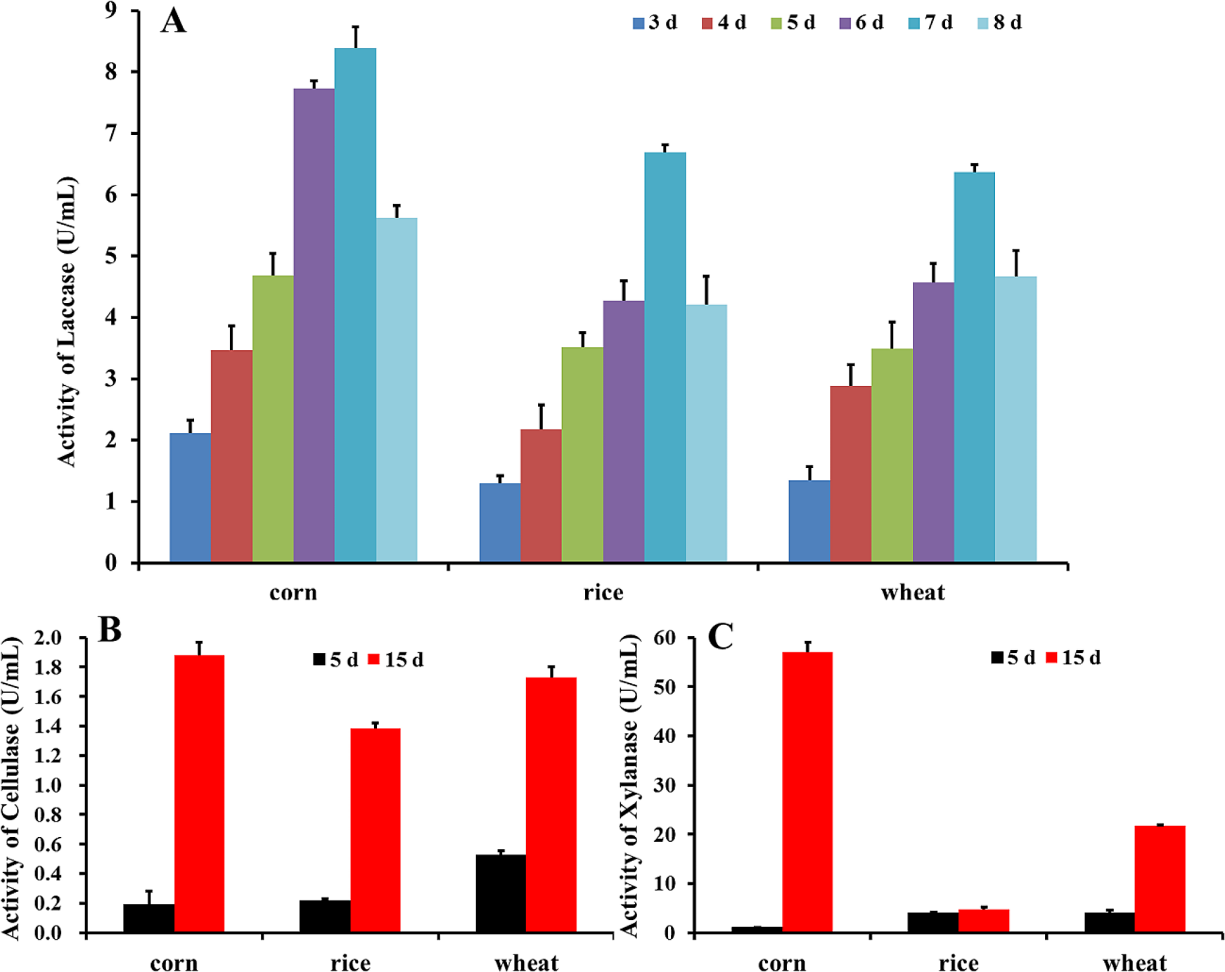
Enzymes activities of GC.u01 during submerged fermentation (SF) with corn stalk, rice straw or wheat straw. **(A)** Lac activity; **(B)** Cellulase activity; **(C)** Xylanase activity

To further study the degradation of crop straw by GC.u01, the enzymatic activities of the three enzymes (Lac, LiP and MnP) were monitored in the pre-treatment via submerged fermentation with corn stalk, rice and wheat straws as substrates were detected. The result showed that Lac activity was generally increased firstly and then decreased in all three kinds of substrates. In all cases, the highest activity of Lac occurred on the 7th day. The maximum Lac activity of three substrates was in corn, up to 8.396 U•mL^− 1^, and then decreased sharply for the substrate gradually exhausted (Fig. 3A). The activities of LiP and MnP were both low and unstable. There were no LiP activity in rice and wheat straws, and the maximum activity was 24.31 U•L^− 1^ on the 6th day in corn stalk. There was no MnP activity in wheat straw, and the maximum activity was 15.22 U•L^− 1^ on the 6th day in corn stalk. The results showed that GC.u01 could secrete mainly Lac to degrade lignin.

Although Lac, MnP and LiP are the major ligninolytic enzymes produced by most fungi, cellulase and xylanase also play synergistic roles in the saccharification of lignocellulosic biomass. The activities of cellulase and xylanase from GC.u01 via submerged fermentation with different crop straws as substrate were also detected. Figure 3 demonstrated the cellulase and xylanase activities with different stalks by GC.u01 through submerged fermentation. All of enzymes showed substantial increase on the 15th day compared with 5th day. The substrate had a significant effect on enzyme activity. The maximum activities of cellulase and xylanase were all with corn stalk and were 1.9 U·mL^− 1^ and 57 U·mL^− 1^, respectively (Fig. 3B and C).

### Effects of biotreatment on major components of crop straw

**Fig. 4:**
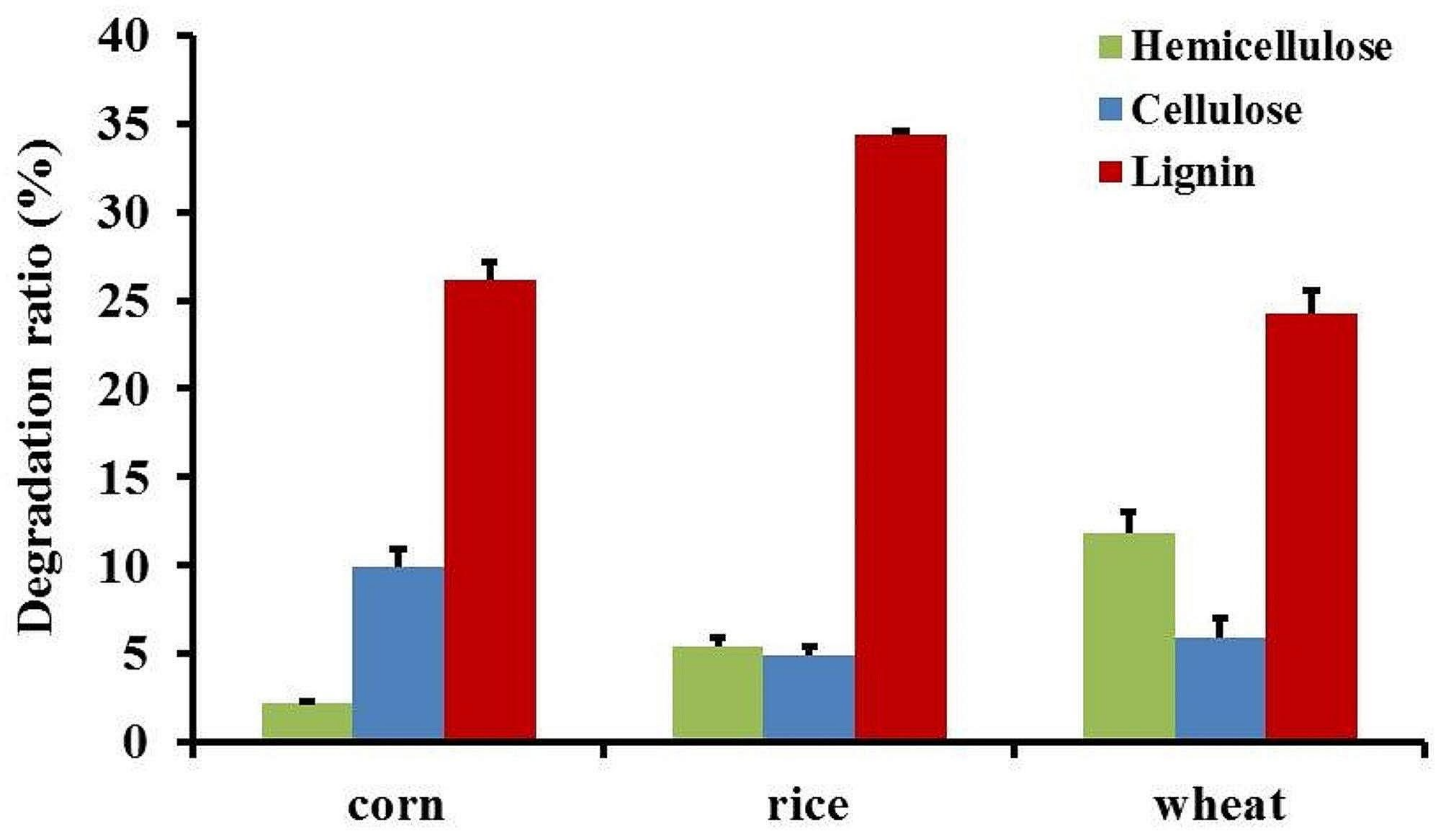
Degradation ratios of crop straws after biotreatment for 15 days

In order to study the degradation degree of crop straw by GC.u01, the contents of lignin, hemicellulose and cellulose in corn stalk, rice and wheat straws before and after fermentation were determined. After pretreated for 15 days using GC.u01, the relative contents of hemicellulose in corn stalk, rice straw and wheat straw were decreased from 27.5 to 26.9%, from 26.0 to 24.6% and from 28.8 to 25.4% (Table 1). The degradation rates were 2.2%, 5.4% and 11.8%, respectively (Fig. 4). The relative contents of cellulose in corn stalk, rice straw and wheat straw were decreased from 31.2 to 28.1%, from 34.5 to 32.8% and from 32.0 to 30.1% (Table 1). The degradation rates of cellulose were 9.9%, 4.9% and 5.9%, respectively (Fig. 4). The relative contents of lignin in corn stalk, rice straw and wheat straw were decreased from 19.5 to 14.4%, from 20.1 to 13.2% and from 21.4–16.2%(Table 1). The degradation rates of lignin were 26.2%, 34.3% and 24.3%, respectively (Fig. 4). It indicated that the isolated *Cerrena unicolor* GC.u01 presented the outstanding degradation capacity of lignin.

**Table 1:** Major components of native and treated crop straws by fermentation after 15 days

Crop straw	Lignin(%)	Hemicellulose(%)	Cellulose(%)
Native corn stalk	19.5 ± 0.4	27.5 ± 0.6	31.2 ± 1.3
**Treated corn stalk**	14.4 ± 0.6	26.9 ± 0.7	28.1 ± 1.2
Native rice straw	20.1 ± 1.1	26.0 ± 1.0	34.5 ± 1.0
**Treated rice straw**	13.2 ± 0.8	24.6 ± 0.9	32.8 ± 1.1
Native wheat straw	21.4 ± 0.4	28.8 ± 0.7	32.0 ± 0.9
**Treated wheat straw**	16.2 ± 0.3	25.4 ± 0.5	30.1 ± 1.0

### Effects of biotreatment on morphological structure of crop straw

**Fig. 5:**
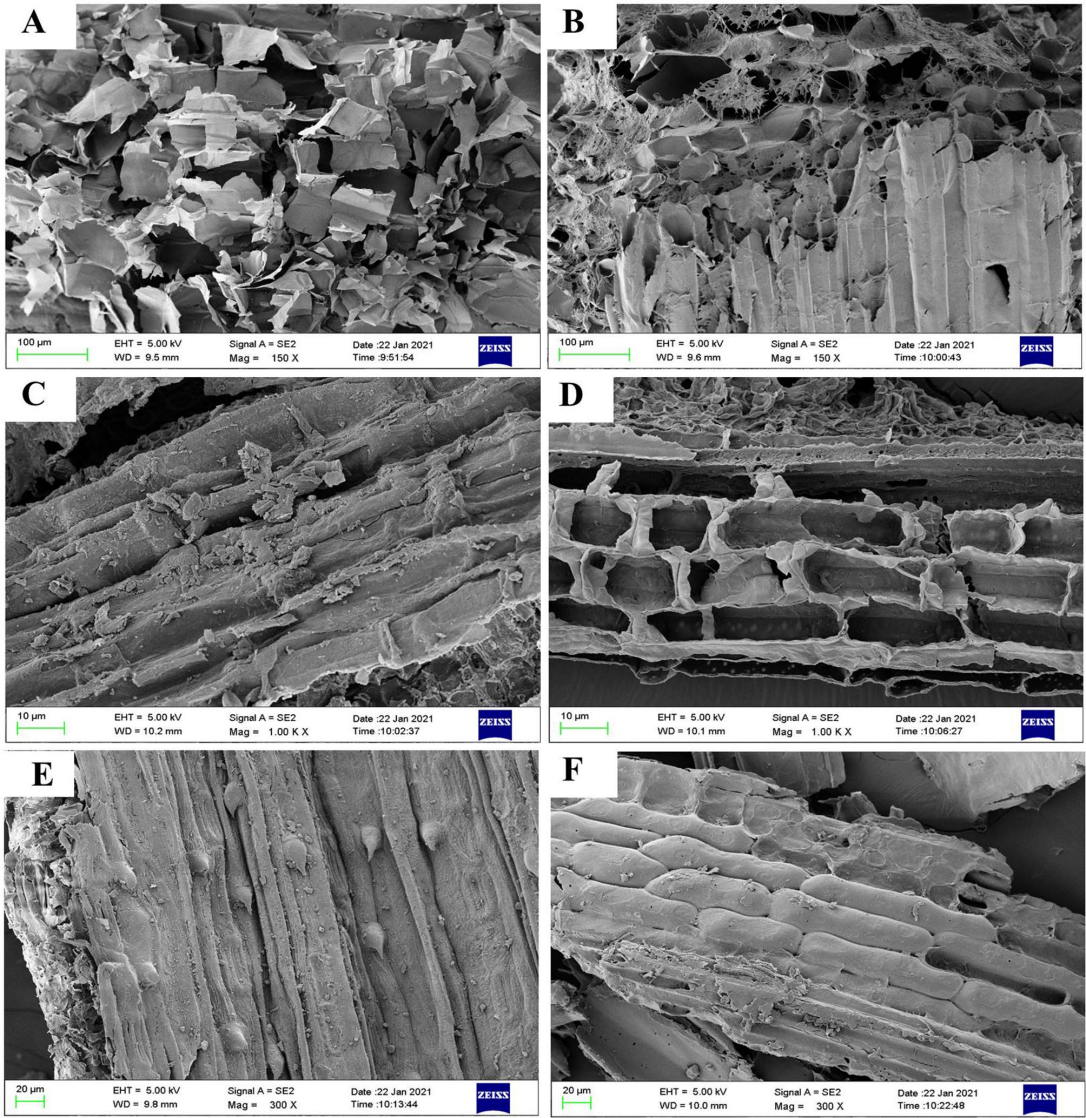
SEM images of corn stalk, rice and wheat straws. **A** and **B**. Corn stalk before and after biotreatment for 15 days; **C** and **D**. Rice straw before and after biotreatment for 15 days; **E** and **F**. Wheat straw before and after biotreatment for 15 days

In order to characterize the effect of GC.u01 fermentation on the morphological structure of corn stalk, rice and wheat straws, the changes of the morphological structure before and after fermentation for 15 days were observed by scanning electron microscopy. Mycelium in the crop straws treated by GC.u01 need be removed. As seen from Fig. 5, the structures of original crop straws were clearly smooth and flat, and the cellulose was closely and orderly arranged (Fig. 5A, C, E). After the fermentation of GC.u01, the surfaces of crop straws were damaged, and the fiber strands appeared crushed, rough and porous (Fig. 5B, D, F), which was caused by the erosion of crop straw lignocellulose through its unique filamentous structure.

### Effects of biotreatment on chemical groups of crop straw

**Fig. 6:**
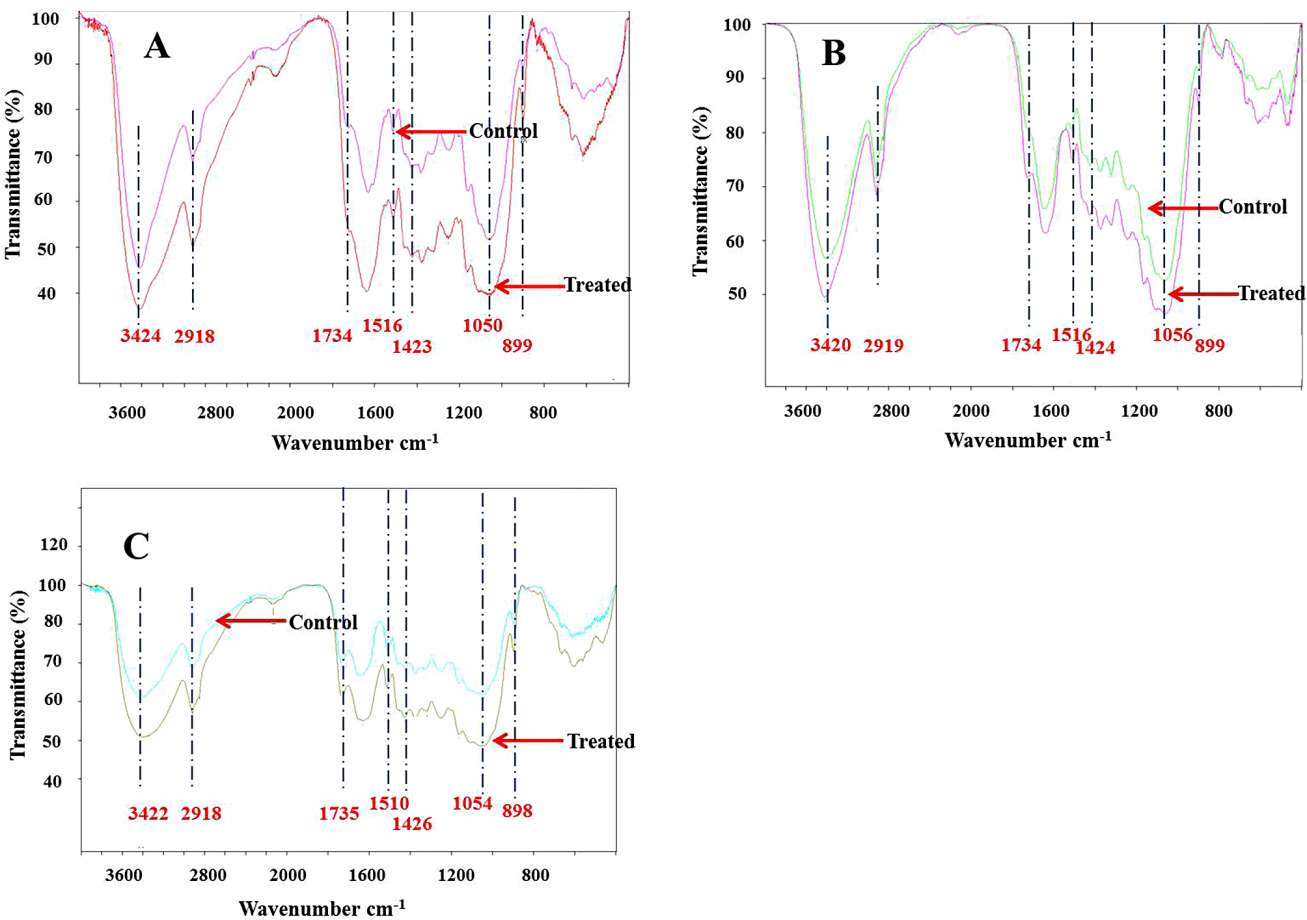
FTIR curves of corn stalk, rice and wheat straws. **(A)** Corn stalk before and after biotreatment for 15 days; **(B)** Rice straw before and after biotreatment for 15 days; **(C)** Wheat straw before and after biotreatment for 15 days. Control stands for untreated sample

In order to further investigate the effect of the pretreatment of GC.u01 on the structure of crop straw, chemical structures were characterized by fourier transform infrared spectroscopy (FTIR). The attribution of infrared characteristic peak were determined (Table 2) (Mou et al. 2014). The result showed that the characteristic peaks of straw before and after degradation for 15 days were significantly different, and the changes were mainly concentrated in 1800 cm^− 1^~400 cm^− 1^(Fig. 6). The bands at around 1734 ~ 1735 cm^− 1^ (C = O conjugates in hemicelluloses) were reduced in treated samples. The peaks at 1510 ~ 1516 cm^− 1^ and 1423 ~ 1426 cm^− 1^ (aromatic skeletal vibrations in lignin) decreased after pretreatment.

**Table 2:** Summary of chemical band assignment by FTIR (Mou et al. 2014)

Band position(cm^− 1^)	Assignment
3400	O-H stretching (polysaccharides/lignin/wax)
2919–2930	C-H (CH_3_ and CH_2_) stretching (polysaccharides/lignin)
1738 − 1734	C = O conjugates (xylans)
1513	C = C stretching and a change in bond angle of the H-C-C of aromatic ring
1425	C-H deformation plus aromatic skeletal vibrations
1040	C-O-C stretching polysaccharides
897	β-glucosidic (polysaccharides)

### Effects of biotreatment on thermal stability of crop straw

**Fig. 7:**
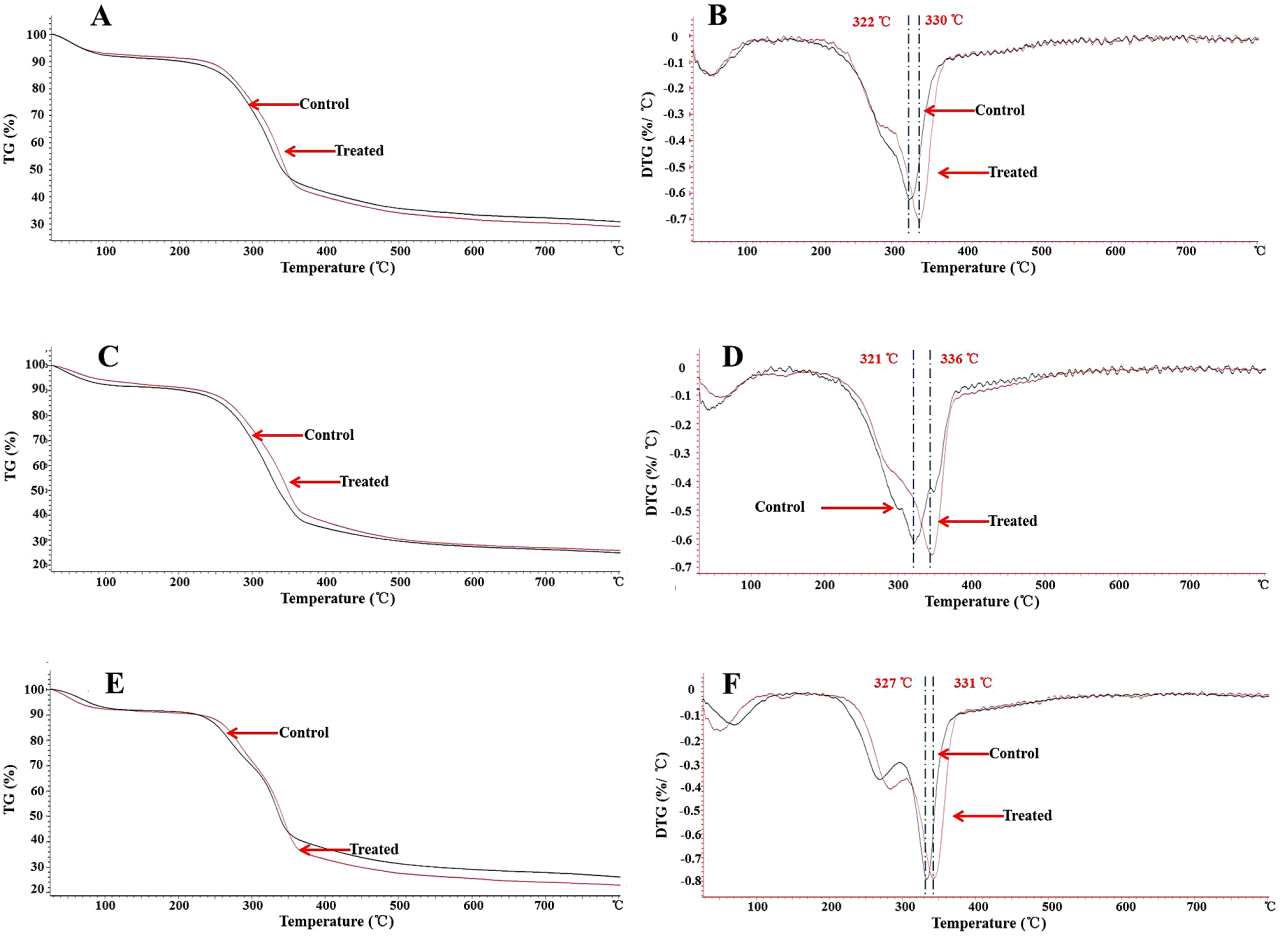
TG and DTG curves of corn stalk, rice and wheat straws. **A** and **B**. TG and DTG curves of corn stalk before and after biotreatment for 15 days; **C** and **D**. TG and DTG curves of rice straw before and after biotreatment for 15 days; **E** and **F**. TG and DTG curves of wheat straw before and after biotreatment for 15 days

In order to further investigate the effect of the pretreatment of GC.u01 on the structure of crop straw, thermal stability was characterized by TGA. With the heating rate of 10 °C/min, the thermal stabilities of crop straw (corn stalk, rice and wheat straws) were determined. The thermogravimetry (TG) and differential thermogravimetry (DTG) carves were showed in Fig. 7. TG curves represented the relationship between crop straw mass change and temperature, and DTG curves represented the decomposition rate of crop straw at different temperatures. The first stage of weight loses at 100 to 180 °C were respectively 9.4%, 9.1%, 8.6% and 8.5%, 8.2%, 9.2% with corn stalk, rice straw, wheat straw before and after pretreatment for 15 days. The treated corn stalk samples had a lower remaining residue (28.8%) at the final temperature than that of the control (30.6%). The treated wheat straw samples also had a lower remaining residue (22.7%) than that of the control (25.9%). The remaining residue of treated rice straw (25.7%) was closed to that of the control (24.8%), but the curve of TG shifted towards a little higher temperature range after fermentation, which was consistent with the change of the peak degradation temperature in the DTG curve.

## Discussion

As one of the major components of lignocellulosic biomass, lignin has been considered as the most abundant renewable aromatic feedstock in the world (Cui et al. 2021). Lignin degradation is the bottlenecks of efficient utilization of biomass. Biological conversion is a promising approach featuring with mild conditions and diversity.

The degradation of lignin by either fungi or bacteria mainly depends on the action of a series of enzymes including Lac, LiP and MnP (Chen et al. 2012; Cui et al. 2021). Zhang et al. (2022) found that the corn stover lignin could be degraded by the three enzymes individually, but the highest percentage of lignin degradation was obtained with a mixture of three enzymes (25.79%). GC.u01 simultaneously secreted three enzymes related to lignin degradation, but the activities of these three ligninolytic enzymes showed significant difference. Lac was the major exoenzyme and had high activity, while the activities of LiP and MnP were only detected on certain days (Fig. 3). The results showed that GC.u01 could secrete mainly Lac to degrade lignin, and this was consistent with *Pycnoporus cinnabarinus* (Eggert et al. 1996a, b). The results showed that GC.u01 could secrete mainly Lac to degrade lignin, and this performance was also agreed with *Pycnoporus cinnabarinus*. Neither lignin peroxidase nor manganese peroxidase in fermentation broth of *P. cinnabarinus* was detected (Eggert et al. 1996a, b). Liu et al. (2013) also found that laccase was the predominant extracellular enzyme among all ligninolytic and hydrolytic enzymes in the extracts from solid phase *Pycnoporus* sp. SYBC-L3 cultivation. Wang et al. (2017) found that the activity of Lac from *Cerrena unicolor* GSM-01 was 2800 U•mL^− 1^ towards ABTS.

Microorganism has decomposition properties for different materials. Different white-rot fungi can degrade 30%~70% of lignin from different lignocellulosic biomasses within 7 ~ 80 days (Li et al. 2022). Ding et al. (2019) found that the lignin degradation rate was 19.05% after 25 days of *P. sajor-caju* treatment to pretreat corn straw. Vasco-Correa et al. (2016) used *Ceriporiopsis subvermispora* to degrdate miscanthus and the lignin degradation was 30% after 28 days. Sugarcane bagasse was treated by *Ceriporiopsis. subvermispora* for 60 days with 48% lignin degradation (Da Silva Machado et al. 2017). It also been reported that the lignin degradation rate of rice straw using *Pleurotus ostreatus* in combination with milling was 34.8% after 30 days (Mustafa et al. 2017). Co-culture pretreatment of strains showed synergistic effects. The degradation rate of lignin by a composite strain of *Phanerochaete chrysosporium, Trametes ersicolor, and Pleurotus ostreatus* was 43.36% which was significantly higher than those of previous studies (Chu et al. 2021). In this study, the result showed that the proportions of lignin content decreased, indicating that GC.u01 had lignin degradation ability. Especially with rice straw, 34.3% degradation rate can be achieved only after 15 days by GC.u01 treatment which took shorter degrading time than other strains although degradation rate was similar with other optimal strains. GC.u01 had certain application potential in lignin degradation.

It was proved that the surfaces of crop straws were damaged and the fiber strands appeared crushed, rough and porous after the fermentation of GC.u01 (Fig. 5B, D, F). The structure of the straw was changed significantly in fermentation process, and the lignocellulosic components which supporting the structure of the straw were effectively degraded. The damage of straw fiber surface structure could increase the accessibility of cellulose and hemicellulose in the subsequent enzymatic hydrolysis process to improve the conversion rate of cellulose. It also predicted that some wastes containing lignin, such as tea residues, wood chips, might be used as both support and nutrient of Lac production by GC.u01. Sun et al. found that after pretreatment with *T.hirsute* YJ9, holes appeared on the surface of corn straw consistent with this study (Sun et al. 2011).

FTIR and TGA analysis further showed that the pretreatment process by GC.u01 had significant effects on the structures of corn stalk, rice straw and wheat straw. The result showed that the characteristic peaks of FTIR straw before and after degradation of GC.u01 for 15 days were significantly different, and the changes were mainly concentrated in 1800 cm^− 1^~400 cm^− 1^ (Fig. 6). The wide absorption bands of 3300–3500 cm^− 1^ were presented as OH stretching vibration signals correspond to carbohydrates (cellulose, hemicellulose, starch and monosaccharide, etc.) and the increase in peaks here indicated that the pretreatment process had exposed cellulose and hemicellulose. The absorption around 2918 cm^− 1^ was attributed to C-H stretching vibration signals (CH3 and CH2 in polysaccharides and lignin) (Mou et al. 2014; Tang et al. 2017a, b). The bands at around 1734 ~ 1735 cm^− 1^ (C = O conjugates in hemicelluloses) were reduced in treated samples, which showed that the removal of hemicellulose after pretreatment (Mou et al. 2014). The peaks at 1510–1516 cm^− 1^ and 1423 ~ 1426 cm^− 1^ (aromatic skeletal vibrations in lignin) decreased after pretreatment indicating that lignin was degraded to a certain extent (Tang et al. 2017a). A wide bands at 1000 ~ 1080 cm^− 1^ were characteristic of C-O-H stretching of primary and secondary alcohols of cellulose and a small bands at 898–899 cm^− 1^ were characteristic of β-1,4-glycosidic linkages in cellulose. The enhancement of these peaks in FTIR spectra of treated samples was due to the increased proportion of cellulose (Long et al. 2012). The thermal properties of lignin in crop straw are closely related to aromatic ring skeleton structure, which could be used as an assessment parameter to evaluate the structural changes of lignin (Ke et al., 2013). It exhibited a higher peak degradation temperature after treatment in all three DTG curves of crop straws. It may be caused by partial removal of hemicellulose and lignin and other components (Sun et al. 2015). This showed that the corn stalk, rice and wheat straws had a high degradation temperature and a high thermal stability after treatment. The degradation rates of crop straws after treatment were higher than that of controls, which may be is attributed to biodegradation, which made the specific surface area of crop straws augmented, more contact points emerged that accelerated the rate of degradation greatly (Zhang et al. 2008). Naresh showed that T_50_ (a measure of the temperature at the level of 50% weight loss) of paddy straw after conventional biotreatment by *Pleurotus florida* was higher than that of control (Naresh et al. 2017). Fubao also showed that the wheat straw after pretreatment had a high thermal stability and low ignition residue due to the partial removal of hemicellulose, lignin and other components (Sun et al. 2015).

In this study, *Cerrena unicolor* GC.u01 with degradation properties for lignin from nature was screened. Qualitative and quantitative tests had shown that GC.u01 had the ability to secrete lignin-degrading enzymes, and the lignin morphology of straws can be changed by depolymerizing lignin. What’s more, GC.u01 could effectively and quickly degrade lignocellulose wastes which can be used as an excellent candidate strain for lignin degradation. In future, the enzyme production mechanism can also be improved through molecular biotechnology, or the way of mixed culture to increase the yield of the enzyme, and enhance the applicability of this strain.

## Data Availability

All data generated or analysed during this study are included in this published article.

## Abbreviations

SEM: Scanning electron microscopy
FTIR: Fourier transform infrared spectroscopy
TGA: Thermal gravimetric analyzer
Lac: Laccase
LiP: Lignin peroxidase
MnP: Manganese peroxidase
ABTS: 2,2’-azino-bis (3-ethylbenzthiazoline-6-sulfonic acid)
TG: Termogravimetry
DTG: Differential thermogravimetry
SF: Submerged fermentation
PDA: Potato dextrose agar
